# *Calpurnia aurea* (Aiton) Benth Extracts Reduce Quorum Sensing Controlled Virulence Factors in *Pseudomonas aeruginosa*

**DOI:** 10.3390/molecules25102283

**Published:** 2020-05-13

**Authors:** Sekelwa Cosa, Jostina R. Rakoma, Abdullahi A. Yusuf, Thilivhali E. Tshikalange

**Affiliations:** 1 Division of Microbiology, Department of Biochemistry, Genetics and Microbiology, University of Pretoria, Private Bag X20, Hatfield 0028, South Africa; u14257662@tuks.co.za; 2 Social Insects Research Group, Department of Zoology and Entomology, University of Pretoria, Private Bag X20, Hatfield Pretoria 0028, South Africa; abdullahi.yusuf@up.ac.za; 3 Department of Plant Sciences, University of Pretoria, Private Bag X20, Hatfield 0028, South Africa; emmanuel.tshikalange@up.ac.za

**Keywords:** antipathogenic, anti-quorum sensing, biofilm formation, cell-to-cell communication, plant extract, molecular docking

## Abstract

*Pseudomonas aeruginosa* is the causative agent of several life-threatening human infections. Like many other pathogens, *P. aeruginosa* exhibits quorum sensing (QS) controlled virulence factors such as biofilm during disease progression, complicating treatment with conventional antibiotics. Thus, impeding the pathogen’s QS circuit appears as a promising alternative strategy to overcome pseudomonas infections. In the present study, *Calpurnia aurea* were evaluated for their antibacterial (minimum inhibitory concentrations (MIC)), anti-quorum sensing/antivirulence (AQS), and antibiofilm potential against *P. aeruginosa.* AQS and antivirulence (biofilm formation, swimming, and swarming motility) activities of plant extracts were evaluated against *Chromobacterium violaceum* and *P. aeruginosa*, respectively. The in vitro AQS potential of the individual compounds were validated using in silico molecular docking. Acetone and ethanolic extracts of *C. aurea* showed MIC at 1.56 mg/mL. The quantitative violacein inhibition (AQS) assay showed ethyl acetate extracts as the most potent at a concentration of 1 mg/mL. GCMS analysis of *C. aurea* revealed 17 compounds; four (pentadecanol, dimethyl terephthalate, terephthalic acid, and methyl mannose) showed potential AQS through molecular docking against the CviR protein of *C. violaceum*. Biofilm of *P. aeruginosa* was significantly inhibited by ≥60% using 1-mg/mL extract of *C. aurea*. Confocal laser scanning microscopy correlated the findings of crystal violet assay with the extracts significantly altering the swimming motility. *C. aurea* extracts reduced the virulence of pseudomonas, albeit in a strain- and extract-specific manner, showing their suitability for the identification of lead compounds with QS inhibitory potential for the control of *P. aeruginosa* infections.

## 1. Introduction

*Pseudomonas aeruginosa* is among the leading causes of Gram-negative infections, associated with nosocomial infections, targeting immune-deficient individuals [1]. This includes chronic lung infections in individuals suffering from cystic fibrosis (CF), burns, acute ulcerative keratitis, skin soft-tissue infections, cancer, AIDS, and urinary tract infections (UTIs), among others [2,3]. *P. aeruginosa* exudes various virulence factors, such as pyocyanin, proteases, and hemolysin, contributing to damaging the host tissue [3]. During the progression of the infection, biofilms form, resulting in persistence, thus making the pathogen difficult to eradicate with antibiotics.

The quorum sensing system (QSS) in *P. aeruginosa* controls the associated virulence factors. The quorum sensing system is a bacterial communication associated with mobilizing and regulating various cellular activities, defense systems, invasion of niches, and formation of communities (biofilms) to survive against changing environments. A biofilm is an aggregation of microcolonies formed by polysaccharides such as Pel; Psl; extracellular DNA; and proteins such as CupA, B, and C fimbriae [4,5]. Biofilms rely on intracellular second-messenger signal molecules, including cyclic dimeric guanosine monophosphate (c-di-GMP) and cyclic adenosine monophosphate (cAMP), which serve as transcriptional regulators that affect many responses and cause phenotype motility and oxidative stress mitigation in the *P. aeruginosa* biofilm. Biofilms are involved in no less than 65% of nosocomial infections and up to 75% of microbial infections occurring in the human body [6].

*Pseudomonas aeruginosa* has the potential to comprise four QSS [7]), identified as lasI/lasR, rhlI/RhlR, *Pseudomonas* quinolone signaling (PQS), and integrated quorum signaling (IQS), which are interconnected and affect each other in a hierarchical way to control virulence factors and the formation of biofilms during infections. Activated lasR can autoinduce the Rhll system, therefore activating an autoinduction feed-forward loop and ensuring the correct ratio of 3-oxo-C12-HSL to C4-HSL signal molecules, which in turn dictates the activation of PQS [8]. Pseudomonas quinolone signaling acts as a link and can induce the rhlI gene responsible for the C4-HSL QS molecule production, which binds to the activation/regulatory protein RhIR.

Two other important virulence factors that *P. aeruginosa* utilizes during infection includes swarming and swimming motility. The production of rhamnolipid controls swarming through rhlAB genes [9]. Rhamnolipid affects the migration of bacteria, together with pili and flagella, depending on nutritional availability, like the utilization of carbon, which determines where the biofilm will develop [10]. *Pseudomonas* spp. produce these rhamnolipids under the control of the Las and RhI systems [11]. Swimming depends on a flagellum and is the movement in liquid or low-viscosity conditions (up to 0.3% agar concentration) [12], whereas swarming is a coordinated translocation of the bacteria across semi or solid surfaces. The difference between swimming and swarming is that swimming requires a functional flagellum but not a QSS nor a biosurfactant [13]. Since these virulence factors contribute to damage on the host tissue, strategies to control them are pivotal.

The usual antibiotic treatment for *P. aeruginosa*-associated infections is inept, due to rising antibiotic resistance. Inhibiting QS through the use of medicinal plants appears to be a vital approach and is purported as a promising antivirulence or antipathogenic agent against bacterial pathogens. Some of these medicinal plants are edible and deemed as safe [14]. However, their potentials are not being explored due to the required high minimum inhibitory concentrations (MIC) needed in order for them to be efficient/active.

The anti-QS (AQS), i.e., antipathogenic/antivirulence mechanism, proposes that these phytochemicals do not kill the pathogen nor put selective pressure but disrupt and inhibit the expression of some important genes required for infections [14]. Furthermore, the majority of the naturally occurring AQS agents already isolated, identified, and characterized are not yet suitable as chemotherapeutic agents due to toxicity, reactivity, and instability [15] and, thus, cannot be used clinically. Thus, the search for potential antivirulence agents continues.

South Africa is rich in culture and plant diversity, and it is home to around 30,000 different plant species, accounting for 10% of the world’s higher plant species [16,17]. This diversity and the long-term relationships that South Africans have with their natural environment mean that they use some of this richness as medicine. Some of the commonly used medicinal plants include *Calpurnia aurea*, *Leonotis ocymifolia,* and *Moringa oleifera*. Moringa possess wound-healing, anti-inflammatory, antioxidant, antimicrobial, antihelminthic, antipyretic, antidiabetic, antihypertensive, lipid-lowering, antifertility, antitumor, hepatoprotective, and antiulcer properties [18]. Health properties of Moringa are attributable to the numerous vital bioactive components it contains, such as vitamins, phenolic acids, flavonoids, isothiocyanates, tannins, and saponins [18]. *Leonotis leonurus*, a Lamiaceae (mint) commonly referred to as “wild dagga” or “lion ear”, is used in folk medicine for the treatment of hypertension, coughs, and headaches. *L. leonurus* is reported to comprise active compounds labdane diterpenes, acyclic diterpenes, iridoid glycosides, alkaloids, dicarboxylic acid, and flavonoids [19]. *Calpurnia aurea* is used as an insecticide to treat lice infestations, coughs, syphilis, leishmaniasis, tapeworm, trachoma, ringworm, scabies, elephantiasis, abscesses, wounds, stomachache, vomiting, headache, and eye diseases [20]. Some of the phytochemical constituents isolated from *C. aurea* include quinolizidine alkaloids, the flavonoids vicenin-2 (6,8-di-β-D-glucopyranosyl-5,7,4′-trihydroxyflavone), butin (7,3′,4′-trihydroxyflavanone), and 3′-hydroxydaidzein (7,3′,4′-trihydroxyisoflavone).

Reports on the antimicrobial activities or use of these plants to treat various ailments are available [18,19,20]. However, none of these reports test the anti-QS or antibiofilm properties of these plants. In effort to validate their use in folk medicine, extracts of these plants (*C. aureus*, *L. leonurus,* and *M. oleifera*) were evaluated for antibacterial and antipathogenic potential. Abilities of the extracts to interfere with biofilm formation and quorum sensing-controlled violacein were also evaluated using in silico molecular docking. However, to limit the scope and focus of this paper, only details of the results obtained from *C. aureus* extracts, which were the most promising, are presented and discussed.

We used the wild-type strain of *Chromobacterium violaceum* (ATCC 12472), a Gram-negative and well-accepted biomonitor strain, in the screening for compounds/extracts with AQS abilities and further antivirulence activities against *Pseudomonas aeruginosa*. *Chromobacterium violaceum* possesses the ability to produce N-Acyl homoserine lactones (AHLs) and can induce quantifiable violacein QS traits. Hence, we measured the AQS ability of the extracts by the significance of their inhibition [21].

## 2. Results

### 2.1. Antibacterial Activities

#### 2.1.1. In Vitro Antibacterial Activities

*Pseudomonas aeruginosa* also produces the blue-green (pyocyanin) and yellowish fluorescent (pyoverdin) pigments on the bacterial media, controlled by *Pseudomonas* QSS; the inhibitory effect of these by the test plant extracts was observed. However, our results indicated that only ethanolic extracts of *Calpurnia aurea* demonstrate antibacterial activity, as observed with clear zones of inhibition ranging from 1.00–20.30 mm. This inhibition was in a concentration-dependent manner as follows: 10.00 mm (1 mg/mL) followed by 15.10 mm (1.5 mg/mL), 17.10 mm (2 mg/mL), and 20.30 mm at 2.5 mg/mL as opposed to the mere inhibition of pigments (pyocyanin/ pyoverdin).

#### 2.1.2. Minimum Inhibitory Concentrations (MIC)

Microbroth dilution assay was used to determine the MICs of selected plant extracts against the planktonic growth inhibition of *P. aeruginosa* ATCC 9721, and results were observed after the color change systems where *p*-Iodonitrotetrazolium violet (INT) dyed the bacterial growth cells red. The MICs against *P. aeruginosa* of the plant extracts from *Calpurnia aurea, Leonotis ocymifolia,* and *Moringa oleifera*, extracted in acetone, ethanol, and ethyl acetate, are shown in Table 1. Minimum inhibitory concentration values ranged from 1.56 to 6.25 mg/mL. Of the nine plant extracts, ethanolic and acetone extracts of *C. aurea* showed potent inhibitory effects, with MIC values of 1.56 mg/mL for both. Although these extracts show effective inhibition against *P. aeruginosa,* these were less potent compared to the positive control ciprofloxacin showing noteworthy antibacterial activity (MIC = 0.04 mg/mL). The extracts of *L. ocymifolia* and *M. oleifera* showed higher inhibitory effects on *P. aeruginosa* with MIC values of =6.25 mg/mL. When the same assay was carried out on the AQS biomonitor strain *Chromobacterium violaceum*, all extracts demonstrated MIC values of 6.25 mg/mL, except for acetone and ethanolic extracts, which showed MIC values of 3.13 mg/mL.

#### 2.1.3. Growth Kinetics of Pseudomonas Aeruginosa Treated with Calpurnia Aurea

To further evaluate the effects of plant extracts on the test pathogen, treatments with *C. aurea* extracts at a sub-MIC concentration of 1 mg/mL at 37 °C for 36 h were made, and results were recorded. As alluded to earlier, *Calpurnia aurea* extracts were chosen due to their potential effects and to narrow the scope and focus of the study.

**Figure 1:**
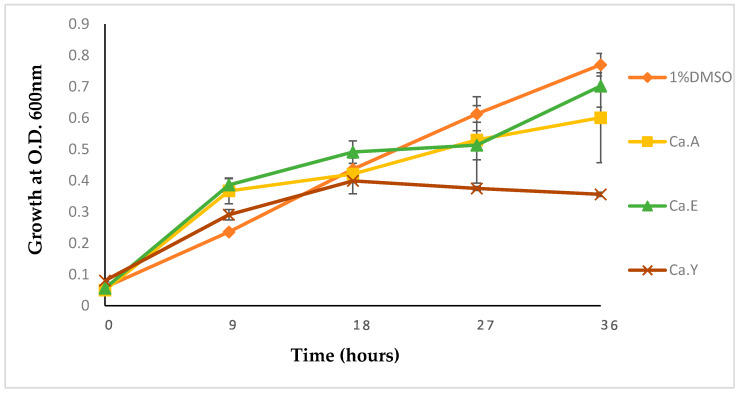
Growth kinetics of *Pseudomonas aeruginosa* ATCC 9721, 1% DMSO-treated, and treatments with different *Calpurnia aurea* extracts prepared at 1 mg/mL incubated at 37 °C for 36 h. The experiments were in triplicates and values presented are means ± standard deviation. Ca.A, Ca.E, Ca.Y—Ca denotes *C. aurea,* A denotes acetone, E denotes ethanol, and Y denotes ethyl acetate extracts. Negative control is the bacteria treated with 1% DMSO. O.D.: optical density.

Inhibitory responses of *P. aeruginosa* were observed when compared with the control test. Bacteria treated with 1% DMSO (negative control) showed a steady exponential growth of *P. aeruginosa* ATCC 9721 over time with an optical density (OD) of 0.78 at 36 h. Inhibition of growth with test extracts ranged from 12–51% with the highest inhibition (51%) observed for the *C. aurea* ethyl acetate extract (Figure 1). All bacteria treated with plant extracts showed a lag phase comparable to the negative control (1% DMSO-treated bacteria). However, steady growths were observed at 18 h, whereas the negative control grew exponentially (Figure 1).

### 2.2. Anti-Quorum Sensing (AQS) Activity of Plant Extracts

#### 2.2.1. Qualitative AQS Activity

Based on the literature [22], violacein pigmentation controlled by QS chemical communications in *C. violaceum* ATCC12472 provides a naturally occurring and readily observable phenotype, without the need for additional substrates, which offers an easy evaluation of the QS inhibition of compounds. The loss of the QS-mediated purple violacein pigment by *C. violaceum* (opaque halo) around the agar wells with plant extracts was recorded as AQS activity. The violacein inhibition (Table 2) showed that *C. aurea* plant extracts inhibited pigmentation in a dose-dependent manner, with zones ranging from 8–12 mm in diameter. None of the other plant extracts showed pigment inhibition.

#### 2.2.2. Quantitative AQS Activity

**Figure 2:**
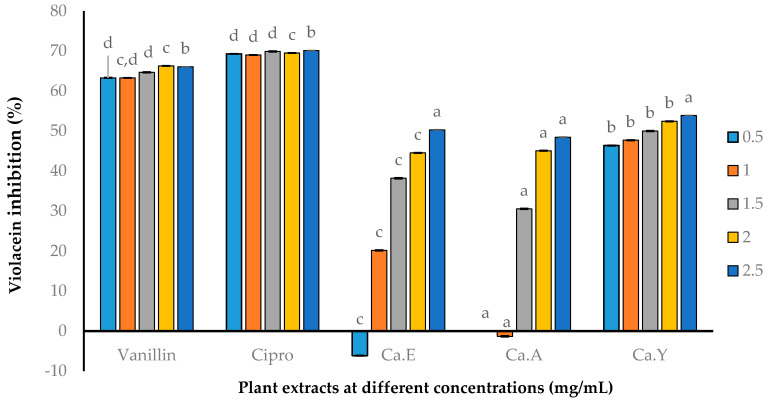
Violacein production by *Chromobacterium violaceum* ATCC 12472 at different concentrations. *C. aurea* extracts: Ca.E—Ethanol, Ca.A—Acetone, and Ca.Y—Ethyl acetate extract. Data presented are mean ± SD, *n* = 3. Different letters represent statistical difference at *p*-value = 0.05 (GLM: generalized linear model and LSD: least significant difference).

Due to the irreproducibility of agar well-diffusion techniques and various limiting factors, we opted for a quantitative violacein inhibition assay. A one-fold dilution (0.50–2.50 mg/mL) was used against the test extracts (*C. aurea* ethanol, acetone, and ethyl acetate). The results obtained demonstrated inhibition in a concentration-dependent manner, with AQS activity of 47.70% at 1 mg/mL for the ethyl acetate extract, whereas the other extracts (ethanol and ethyl acetate) were significantly lower at this concentration. *C. aurea* ethyl acetate, followed by the ethanol extract (Ca.Y), were more potent compared to the acetone extracts. Vanillin (a known AQS agent) and ciprofloxacin were observed with significant AQS activities at all concentrations (Figure 2).

### 2.3. GC-MS Analysis of Calpurnia Aurea Extract

**Figure 3:**
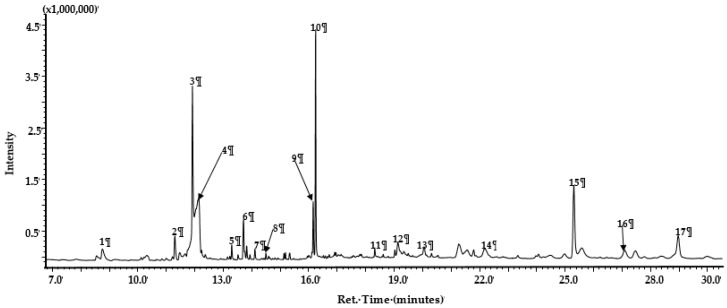
Representative total ion chromatogram (TIC) of the ethanolic extract of *Calpurnia aurea*. Peak numbers and their identities correspond to those in Table 3.

GC-MS profiling of *Calpurnia aurea* extracts (Table 3 and Figure 3) showed an abundance of compounds in the following chemical classes: organic acids and their esters (32.06%), alcohols (29.83%), sugars (15.76%), aldehydes (4.95%), and hydrocarbons (1.87%), with steroids (1.06%) as the minor components. Specific major components in the profile include organic acid ester 1,4-benzenedicarboxylic acid diethyl ester, methyl mannose, pentadecanol, and eicosanol. The profile also contains the steroid beta-H-pregna and aldehydes octadecanal and tetracosanal (Table 3 and Figure 3).

### 2.4. In Silico Molecular Docking of Compounds Identified by GC-MS

**Figure 4:**
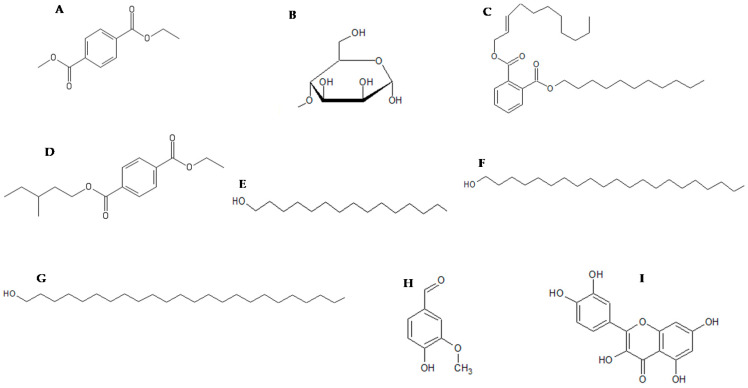
2-dimensional structures of the compounds identified from the *Calpurnia aurea* extract used in molecular docking against the *Chromobacterium violacein* CviR protein. (**A**) = Dimethyl terephthalate, (**B**) = Methyl mannose, (**C**) = Phthalic acid undecene-undecyl ester, (**D**) = Teraphthalic acid, ethyl isobutyl ester, (**E**) = Pentadecanol, (**F**) = Eicosanol, (**G**) = Tetracosanol, (**H**) = Vanillin, and (**I**) = Quercetin. Vanillin and quercetin served as positive anti-quorum sensing (AQS) controls.

**Figure 5:**
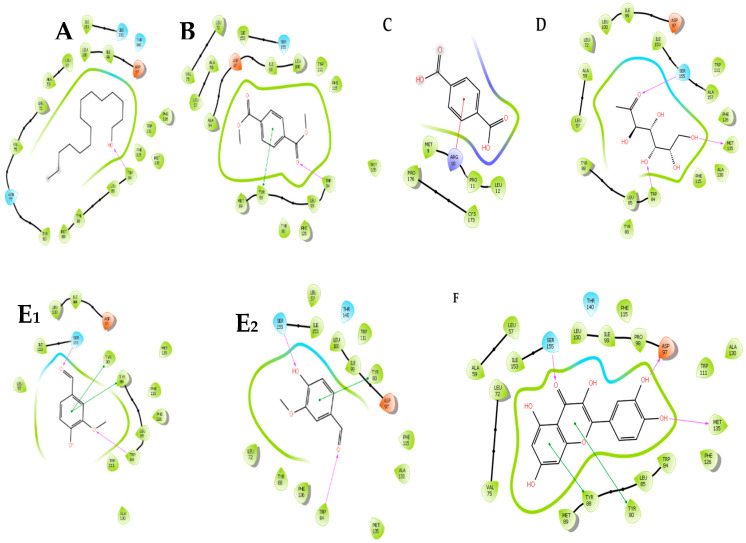
Interaction and binding of selected test compounds against the *C. violaceum* CviR protein active site. The negatively charged protein residues are indicated in red, polar residues are in cyan, hydrophobic residues are shown in parrot green, hydrogen interactions (H-bonds) are presented as pink/purple arrows, *pi*–*pi* stacking is shown as a green line, and the *pi* cation as a red line. (**A**)—Pentadecanol, (**B**)—Dimethyl terephthalate, (**C**)—Terephthalic acid, (**D**)—Methyl mannose, (**E_1_**,**E_2_**)—Vanillin, and (**F**)—Quercertin.

In silico molecular docking of compounds identified from the GC-MS profile (Figure 4) of the ethanolic extracts of *C. aurea* was conducted to substantiate the potential AQS activity observed in the violacein inhibition assay in order to confirm the binding affinity of the molecules to the CviR protein of *C. violacein* (AQS biomonitor strain) (Figure 5 and Table 4). Of the 17 compounds identified, only eight were selected and used in the in silico molecular docking. The compounds (Figure 4) were chosen on the basis of their abundance in the plant extract (see Table 3 and Figure 3). The pentadecanol hydroxyl group binds to the amino acid (a.a) TRP84 (docking score = −3.758, Figure 4A and Figure 5A), whereas, with dimethyl terephthalate, the carbonyl group showed a hydrogen bond interaction with TRP84, and a *pi*–*pi* interaction was observed with the benzene ring and TYR88 (docking score = −5.486, Figure 4B and Figure 5B). Terepthalic acid showed a *pi* cation interaction ARG 10 residue and an improved docking score of −6.186 (Table 4) compared to other test molecules (Figure 4F and Figure 5C). Methyl mannose presented a number of binding poses; however, the best interaction was with a docking score of −7.000 (Table 4). Vanillin (Table 4 and Figure 5E_1_,E_2_) and quercetin (Figure 5F) were included as positive AQS compounds. Quercetin shows the best binding interaction with the CviR protein active site with a docking score = −10.613, while vanillin exhibited −4.880 (comparable to our test compounds, Table 4).

### 2.5. Effect of Plant Extracts on Antiadhesion and Biofilm Eradication

The biofilm formation strength of *P. aeruginosa* ATCC 9721 was assessed using a crystal violet dye assay, in which glass tubes were used to confirm the bacterial adherence to the glass surface and pigment immensity. Results showed the stimulation of the biofilm inhibition upon the addition of *C. aurea* ethanolic extract, as observed by the strong crystal violet pigment retained on the glass tube.

**Figure 6:**
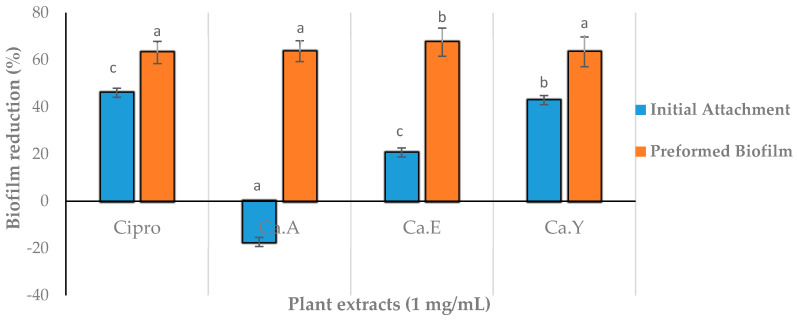
Antibiofilm activities of *Calpurnia aurea* extracts at 1 mg/mL against *Pseudomonas aeruginosa*. Ca.A—Acetone, Ca.E—Ethanol, and Ca.Y—Ethyl acetate extract. Data presented as mean ± standard deviation of three replicates, *n* = 3. Different letters represent statistical differences at *p*-value = 0.05 (GLM and LSD).

Antiadhesion (initial attachment) and biofilm eradication assays against *P. aeruginosa* ATCC 9721 treated with all *C. aurea* plant extracts are shown in Figure 6. Acetone extracts of *C. aurea* showed an adhesion promotion with other plant extracts, reducing the biofilm production up to ±42% (Figure 6). Negative percentage biofilm eradication observed with acetone extracts suggests a stimulation originating from the presence of the secondary metabolites utilized as nutrients for the growth and formation of the biofilm. Highest percentage inhibition against *P. aeruginosa* ATCC 9721 was in the initial attachment inhibition assay at ±20% for the *C. aurea* ethanol extract (Figure 6). The inhibition of preformed biofilm by all three *C. aurea* were observed to be more potent, with up to 60% biofilm reduction as compared to the initial attachment.

#### Confocal Laser Scanning Microscopy (CLSM) Observation of Biofilm Inhibition

**Figure 7:**
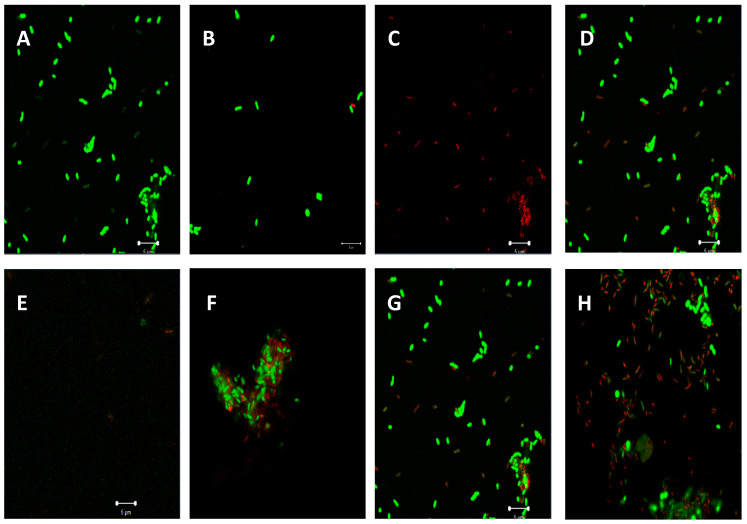
Observation of *P. aeruginosa* cells treated and untreated with test extracts of *C. aurea* ethanol, acetone, and ethyl acetate using confocal laser scanning microscopy (CLSM). (**A**)—*P. aeruginosa* ATCC 9721 cells treated 1% DMSO, (**B**)—Cells treated with *C. aurea* ethanol extract, (**C**)—image showing dead cells only treated with *C. aurea* ethanol extract, and (**D**)—Combination of live and dead cells treated with *C. aurea* ethanol extract. (**E**)—Cells treated with ciprofloxacin (0.06 mg/mL). (**F**–**H**) represent cells treated with ethanol, acetone, and ethyl acetate extracts, respectively. Live cells staining appear green, while dead cells show red fluorescence.

Inhibition were assessed at a 1-mg/mL MIC concentration, and the cell initial attachment showed more inhibition as compared to the preformed biofilm (Figure 7). The negative control (bacteria treated with 1% DMSO, Figure 7A) had more living cells compared to the positive control (ciprofloxacin at 0.06 mg/mL) (Figure 7E). Samples treated with ciprofloxacin (Figure 7E) showed an absence of intact bacterial cells as proof of the deterioration of cells with a fraction of green cell (live cells) remains. Figure 7B,C were compared to show a representation of live cells to the dead cells, respectively. Among the three extracts (Figure 7F–H), the ethyl acetate extract appeared more effective (Figure 7H).

### 2.6. Effect of C. aurea Extracts on Swarming and Swimming Motility

**Figure 8:**
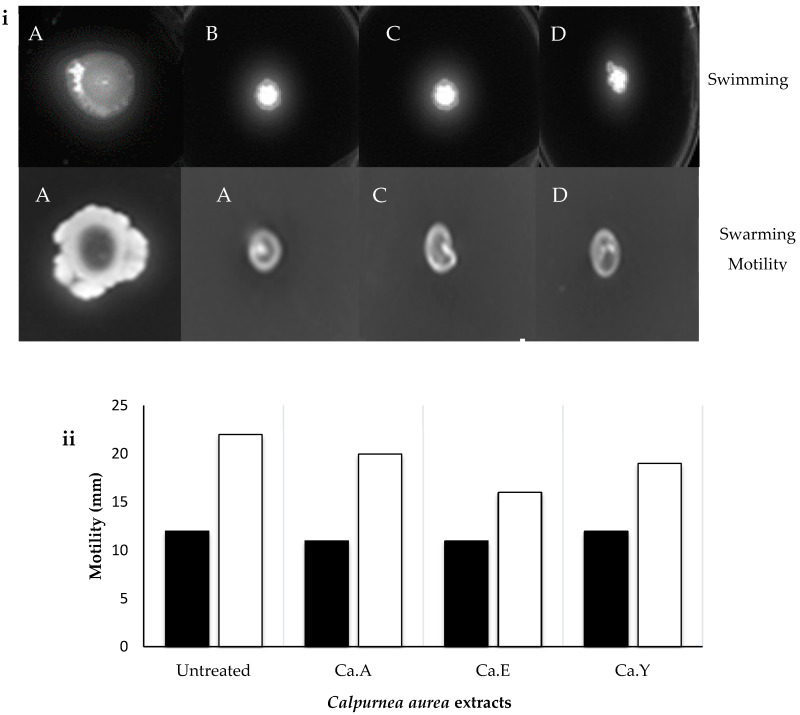
Representative images (**i**) and bar graph (**ii**) of *P. aeruginosa* (ATCC 9721) swimming (closed bars) and swarming (open bars) motility in the presence of various plant extracts (1 mg/mL) after 24 h at 37 °C incubation. Top row: swimming motility and bottom row: swarming motility. (**A**) negative control (untreated bacteria), and (**B**–**D**) represents *P. aeruginosa* treated with *C. aurea* ethanol, acetone, and ethyl acetate, respectively. Ca.E—Ethanol, Ca.A—Acetone, and Ca.Y—Ethyl acetate extracts. Data presented as averages.

When swarming and swimming *P. aeruginosa* were treated with 1 mg/mL MIC of each *C. aurea* extract, the pathogen showed susceptibility to the treatment observed by a limited/restricted motility (Figure 8A–D). Ethanolic, acetone, and ethyl acetate extracts of *C. aurea* all reduced the swarming motility of *P. aeruginosa* significantly compared to untreated cultures (Figure 8iA–D and ii). Similarly, a significant reduction (Figure 8iA–D and ii) in swimming was observed when *P. aeruginosa* was treated with *C. aurea* ethanol, acetone, and ethyl acetate extracts.

## 3. Discussion

Plant material in the form of crude extract infusions, decoctions, or tinctures are used in traditional medicine for infectious diseases therapy. These simple medicinal preparations are often effective due to their active chemical constituents. However, their efficacy and mechanisms of action are often not validated scientifically [23]. The most commonly used organic solvent for herbal medicine manufacture is ethanol, as the final products are safe for internal use by consumers [23]. Hence, nine extracts of *Calpurnia aurea; Leonotis ocymifolia;* and *Moringa oleifera* (each extracted with acetone, ethanol, and ethyl acetate) were subjected to biological assays in validation of some of their antibacterial activities, AQS activities, antivirulence, and/or antibiofilm potential and in silico docking potential used in traditional African medicine. Of the nine extracts made from the three plants, *Calpurnia aurea* extracts were more effective, displaying improved antibacterial activities from the results obtained in the qualitative screening (agar well-diffusion assay) and MIC values (Table 1), particularly when compared to the extracts from *Leonotis ocymifolia* and *Moringa oleifera*. Based on these preliminary screening assays, four different concentrations ranging from 1–2.5 mg/mL showed significant zones of inhibition in a concentration-dependent manner.

Based on the MICs, *C. aurea* extracts were more effective compared to *Leonotis ocymifolia* and *Moringa oleifera*, mirroring the results obtained in the preliminary screening for antibacterial activity. The results obtained either display the antibacterial inefficiency of both *M. oleifera* and *L. ocymifolia* or emphasizes the resistance of the bacteria to antimicrobial treatments using these plant extracts. In this context, *Pseudomonas* pathogens are known for their difficult nature and a serious therapeutic challenge with regards to a response to antibiotic treatments. Even more challenging is the ability of *P. aeruginosa* to develop resistance in the course of the therapy. This is due to the pathogen either acquiring resistance genes on mobile genetic elements (i.e., plasmids) or through mutational processes that alter the expression and/or function of chromosomally encoded mechanisms. In both scenarios, the severity is limiting the therapeutic options for the treatment of serious infections [24].

When the antibacterial test of essential oils extracted from *L. ocymifolia* was investigated [25] against *Pseudomonas aeruginosa*, amongst other test bacteria, the MIC was reported as 1.25 mg/mL, which this is significantly lower than the 6.25 mg/mL reported in the present study. However, this may not be a good comparison, as essential oils and solvent extracts may have different antibacterial mechanism effects on the pathogen. When the aqueous-ethanolic extract of *Leonotis nepetifolia* was assessed for antibacterial activity on selected bacterial strains, including *Pseudomonas aeruginosa,* the observed MIC value was 800 µg/mL [26], which is comparatively higher than the findings here (MIC ≥ 6.25 mg/mL) for the acetone, ethanol, and ethyl acetate extracts. Our approach of using different solvents was more favorable for exploring these plants and their antibacterial properties, as these solvents differ in their abilities and efficiencies in extracting active components, and they provide more avenues for upscaling against the use of aqueous extracts that are restricted to small-scale usage.

Both acetone and ethanolic extracts of *Moringa oleifera* were reported to show no inhibition on *Streptococcus faecalis, Bacillus pumilus, Klebsiela pneumonia,* and *Bacillus cereus*, including *P. aeruginosa* [26,27]. The obtained findings revealed *P. aeruginosa,* including *Escherichia coli* and *Salmonella enteritidis* (IH), were resistant to all treatments using ethanolic leaf extracts from Moringa. Our findings here are incongruent with the findings that chloroform and aqueous extracts of Moringa have no antibacterial activity against *P. aeruginosa* [28].

The results of the present study for MICs are deemed not noteworthy, with MIC values > 1 mg/mL. However, when compared to the literature [29], our reported MIC values are significantly lower—in particular, those for *C. aurea* acetone and ethanolic extracts. Previous reports on the inhibitory effects of *C. aurea* are contradictory, with methanolic leaf extracts reported to inhibit the visible growth of *P. aeruginosa* with an MIC of 125 mg/mL [30], while no antibacterial or antioxidant activities were reported for the leaves and stem extracts [31]. This inactivity and higher MICs may be attributed to a resistance to penetration by the phytochemical extract due to the protective outer membrane on the cell walls of *P. aeruginosa* and other Gram-negative bacteria. Alternatively, this may also be attributed to the ability of *P. aeruginosa* to actively efflux the compounds from the cell and alter its phytocompound target.

Due to the efficacy of ethanolic extracts from *C. aurea* against *P. aeruginosa*, we did focus this study on those and used them for further bioassays. Moreover, we found no report in the literature on their modulatory effects or antipathogenicity (AQS) on any pathogen. In most cases, AQS agents demonstrate poor MIC activities but might have the ability to obstruct the bacterial cell-to-cell communication facilitated through the QS system [14].

To corroborate the antibacterial activity of *C. aurea* extracts on *P. aeruginosa*, the pathogen was treated with the different test extracts at 1 mg/mL (37 °C), and the growth profile was monitored over a period of 36 h. The initial steady growth observed could be explained by the ability of *P. aeruginosa* to express or exhibit innate resistance because of the low permeability of the outer membrane (OM) [31]. The later decrease in bacterial growth could be attributed to a minute treatment able to penetrate the OM, thereby beginning a slow release of the bacterial cell components, leading to cumulative cell death with time. Preferably, for the test extracts to serve as AQS and/or antipathogenic agents, they should exhibit less inhibition to the bacterial growth, as the hypothesis of antipathogenic drugs should display no bactericidal effect on the microbes, except to hinder or disrupt the virulence factors.

Screening for AQS activities has relied on tempering with various virulence factors of the pathogen. At present, *C. violaceum* (*Chromobacterium violaceum* ATCC 12472, pigmenting strain that overproduces C6-AHL, the autoinducer), amongst others, is a well-accepted pathogen used in screening for anti-QS agents or as a biomonitor strain. This is due to the fact that *C. violaceum* produces violacein (purple pigment), which is a QS-dependent factor controlled by the CviR-QS system [32]. Any effect on the violacein pigment due to the test extract treatment suggests tempering with the QS system of the pathogen. Hence, what we have shown here is, primarily, the qualitative violacein inhibition, as evident by the diameters of the opaque zones around the wells loaded with the plant extract. Qualitatively, AQS activities were dose-dependent, with *C. aurea* acetone followed by ethanolic extracts showing a significant opaque zone of inhibition at a concentration of 1 mg/mL. In studies of anti-QS activity using disc diffusion [33] containing 3 mg/mL of plant extracts, ethanolic crude extracts of *Laurus nobilis* leaves, flowers, fruits, and bark showed anti-QS with 17, 24, 15, and 19 mm zones of inhibition, respectively. Floral and leaf extracts of *Jasminum sambac*, *Rosmarinus officinalis* (L), *Populus alba* (L), and *Populus nigra* (L) exhibited weak anti-QS activities with zone of inhibitions ranging from 8–10.5 mm [34]. In a recent study [14], extracts of *Glycyrrhiza glabra*, *Apium graveolens*, *Capsicum annuum,* and *Syzygium anisatum* demonstrated good anti-QS activities, yielding opaque halo zones ranging from 12 to 19 mm-diameters at sub-minimum inhibitory concentrations (0.35–4.00 mg/mL).

As reported by Cosa et al. [14], a qualitative agar well-diffusion assay is not an appropriate technique to ratify the AQS potentials of plant extracts. Hence, quantitative analyses are recommended as the standard to reduce discrepancies between qualitative and quantitative approaches. Here, when we monitored AQS activities quantitatively, a similar trend of dose-dependent effects correlating the qualitative AQS results were observed between the concentrations 1–2.5 mg/mL. Based on documented studies, various plant extracts show different degrees of AQS activities against *C. violaceum* at different concentrations. Abraham et al. [35] reported a maximum of 88% inhibition in violacein production at the concentration of 2 mg/mL when *Capparis spinose* extracts were assessed quantitatively for their violacein inhibition. Vasavi et al. [36] reported AQS of *Centella asiatica* against *C. violaceum* 026 and ATCC 12472 at 100 mg/mL, with more than 50% inhibition in violacein production observed, while a complete inhibition occurred at 300 mg/mL.

Cosa et al. [14] advocates that, for compounds with AQS potentials to demonstrate bactericidal effects or kill bacteria, their effectiveness should be demonstrated at sub-MIC concentrations. This theory hypothetically suggests the bacteria should be less likely to instigate resistance towards the candidate compounds. Additionally, the compounds must possess chemical stability, negligible toxicity, low molecular weights, and higher degrees of specificity to signal receptors [14]. Furthermore, as the mechanism of action of the plant extracts in this case was not known, we profiled ethanolic extracts of *C. aurea* extracts using GC-MS in order to identify the compounds present that could be responsible for its potency against *P. aeruginosa*. This will give an idea of whether the mode of action is due to the ability to bind onto the CviR protein site of the signaling molecule N-hexanoyl-L-homoserine lactone (C6-HSL). Findings revealed 17 compounds present in the extract—Of which, the eight most abundant (pentadecanol, dimethyl terephthalate, phthalic acid, terephthalic acid, eicosanol, tetracosanol, dodecyl phthalate, and methyl mannose) were selected and used in molecular docking studies (Figure 4) against the binding site of the CviR protein of *C. violaceum*.

Emran et al. [37] showed in silico docking as a potential technique to validate in vitro findings. In silico molecular docking experiments of chemical compounds present in the *Calpurnia aurea* extract validated the AQS activities obtained from our in vitro experiments (due to less and/or no bactericidal effects observed and the AQS potential). Four molecules: pentadecanol, dimethyl terephthalate, terepthalic acid, and methyl mannose showed a binding affinity to the CviR protein. Hence, these compounds may be acclaimed for their AQS activity. The docking scores (Table 4) indicate and confirm the potential AQS activity observed with the plant extracts, although further studies using individual compounds from the extracts are required in order to further validate these findings.

Methyl mannose, one of the most abundant components in the extracts of *C. aurea,* showed a number of binding interactions (Figure 5D), the best being with a docking score of −7.000 kcal/mol, which equated to several interactions on the amino acid residues. It also shows the least binding affinity in comparison to the other molecules (Figure 5), having the best docking conformation [37]. Thereafter, it was followed by terephthalic acid and dimethyl terephthalate, with −6.186 kcal/mol and −5.486 kcal/mol molecular docking scores, respectively. The findings showed an improved docking score compared to the positive control, vanillin (−4.880 kcal/mol). On the other hand, quercetin included as a second positive AQS agent, chosen for its potent docking efficiency to the CviR protein, had an observed docking score of −10.613 kcal/mol. The methyl mannose carbonyl group formed a hydrogen bond with SER155, hydroxyl group with MET135, and another with TRP84. All three molecules showed binding at active sites with TRP84, except terephthalic acid.

These findings corroborate those of the molecule’s interactions with the receptor protein, with methyl mannose having better interactions after quercetin. Pentadecanol showed the least binding interactions (TRP 84). The observed trend cannot be justified with the AQS in vitro assay, as the extracts possess all molecules. However, this data further confirms that the *Calpurnea aurea* extract possesses molecules with the potential to interact with the biomonitor strain receptor protein associated with their QS system.

The quorum sensing system (QSS) in *P. aeruginosa* ATCC 9721 influences and controls the biofilm formation, allowing the attachment of cells onto its surfaces. Biofilms are a complex polymetric matrix of microbial communities, mostly found attached on surfaces, rendering microbes inside the matrix complex able to eradicate antibiotic treatments [38]. Based on our findings, *P. aeruginosa* showed some resistance to treatments with the *C. aurea* plant extracts, with ethanolic and ethyl acetate extracts showing only mild interferences in the initial biofilm formation, while acetone extracts contributed to the promotion of the biofilm. However, the acetone extracts showed a moderate-significant biofilm formation eradication. In both the inhibition of the initial attachment and preformed biofilms assessed, interference with the biofilm formation were observed for all plant extracts. This shows that the extracts could be inhibiting the expression of virulence factors and altering the QS mechanism, thus creating unfavorable conditions for the bacteria [39]. An analysis of the biofilm inhibition by CLSM shows interference and disruption of the biofilm formation and cell structure due to the treatments with the plant extracts. Disruptions and fractioning of damaged cell membranes/dead cells were clearly visible in comparison to the negative control (Figure 8).

The swarming and swimming motility of *Pseudomonas aeruginosa* infers a pivotal role in the initiation, maturation, and maintenance of the biofilm architecture [33]. Thus, prohibiting the motility of bacterial pathogen may contribute to hindering its pathogenicity. We found in this study that both swarming and swimming were hindered by the presence of all test extracts when compared to the negative control (pathogen treated with 1% DMSO). The treatment showed a significant decrease of swarming and a reduction in the diameters of the swim zones. Similar findings were reported by Al-Yousef et al. [33], where *Allium cepa* L (onion peel ethyl acetate fraction (ONE)) effectively reduced the swarming motility of *Pseudomonas aeruginosa* (PAO1) by 22–61%. Based on these findings, tempering with the pathogen’s migration suggests the potential to hinder its ability to form biofilms, a key factor hindering treatments with many antibiotics.

## 4. Materials and Methods

### 4.1. Plant Materials and Extractions

Leaves of *Calpurnia aurea*, *Leonotis ocymifolia,* and *Moringa oleifera* were collected at Manie van der Schijff Botanical Garden (University of Pretoria, Pretoria, South Africa) in March 2018. The plants were identified by Jason Sampson (curator) and stored at the Department of Biochemistry, Genetics and Microbiology, University of Pretoria, Hatfield, South Africa. Plant materials were air-dried and then crushed to a fine powder using a pestle and mortar. Fifteen grams (15 g) of each powdered material were soaked in 350 mL of ethanol, acetone, and ethyl acetate with agitation using a rotatory shaker at a speed of 139 rpm for 7 days. The extracts were filtered through Whatman (No.1) filter paper. Thereafter, filtrates were evaporated using a rotatory evaporator at 45 °C under reduced pressure. Extracts were then transferred to preweighed vials and dried in a fume hood cabinet. Dried plant extracts were later redissolved in 1% dimethyl sulfoxide (DMSO) to the required concentration for the biological assays.

### 4.2. Culturing and Maintenance of Microorganisms

Bacterial strains of *Pseudomonas aeruginosa ATCC* 9721 and *Chromobacterium violaceum* ATCC 12472 were used to evaluate the antibacterial and AQS activities of the plant extracts. The bacterial strains were cultivated in Mueller Hinton (MH) and Luria Bertani (LB) media and incubated at 37 °C and 30 °C for *P. aeruginosa* and *C. violaceum*, respectively. For the maintenance of the bacterial strains, glycerol stock cultures of each organism were prepared and kept at −80 °C until required.

### 4.3. Antibacterial Activity

#### 4.3.1. Minimum Inhibitory Concentration (MIC)

The broth dilution method was used to determine the minimum inhibitory concentration (MIC) of the plant extracts, as described by Eloff [40], with a slight modification. A stock concentration (25 mg/mL) of each plant extracts was prepared. A volume of 100 μL of MH broth was transferred to each well of a 96-well microtiter plate. This was followed by placing 100 μL of each plant extract (in triplicate) into the first row of microtiter plates. Serial dilutions were prepared in the direction from A to H, resulting in decreasing concentrations over the range 6.25–0.049 mg/mL. Subsequently, 100 μL of the bacterial culture of *P. aeruginosa* (OD 600 nm = 0.08–0.1) was transferred into each well. Each plate was prepared with a set of positive and negative controls. Ciprofloxacin was used as the positive control at a concentration of 1 μg/mL, while 100 μL of 1% DMSO was used as the negative control. Once prepared, the plates were sealed with adhesive and incubated at 37 °C for 24 h. After incubation, 40 μL of a 0.20 mg/mL solution of p-iodonitrotetrazolium violet (INT) was added to each well and incubated for 1 h. The MIC value for each extract was visually assessed and recorded. The minimum concentration of plant extracts at which there was no visible growth of test strain was regarded as the MIC value. The antibacterial assay was carried out in triplicates, while the experiments were replicated twice.

#### 4.3.2. Growth kinetics of *Pseudomonas aeruginosa*

*Pseudomonas aeruginosa* ATCC 9721 cells were grown in a Mueller Hinton (MH) broth medium in the presence or absence of the different plant extracts at 1 mg/mL sub-MIC concentration using 96-well microtiter plate with shaking at 120 rpm. The plates were read using a xMark microplate spectrophotometer (Manti Lab MT-137A, Haryana, India) at 600 nm every 9 h for 38 h to measure the optical density. Growth curves were extrapolated to determine the effect of the extract on the test pathogen with time.

### 4.4. Anti-Quorum Sensing (AQS) Activity

#### 4.4.1. Qualitative AQS Activity

Agar well-diffusion assay [22] was performed to evaluate the anti-quorum sensing (AQS) potential of each plant extract. A 3 µL volume of each extract was loaded onto a sterile disk (6 mm-diameter), placed onto prepared LB agar plates, and swabbed with standardized overnight cultures of *C. violaceum* and *P. aeruginosa* (adjusted to 0.08–0.1 at 600 nm). Ciprofloxacin and 1% DMSO were used as positive and negative controls, respectively. The plates were then incubated at 30 °C and 37 °C, respectively, for 24 h. Following incubation, plates were evaluated for the inhibition of violacein production (a creamy-white, opaque halo) around the well. The zones (diameter) of inhibition were measured using a processing program in Image J 1.52a software (Java, National Institutes of Health, Rockville Pile, MD, USA). The diameter of the inhibition zone was interpreted as follows: susceptible (S) = 15 mm, intermediate (I) = 11–14 mm, and resistant (R) = 10 mm [14].

#### 4.4.2. Quantitative AQS Activity

Nine plant extracts were evaluated for their ability to inhibit the violacein production of *C. violaceum*, as described by Choo et al. [41], with slight modifications. A volume of 100 µL of each plant extract with varying concentration (0.50–2.50 mg/mL) was pipetted into individual Eppendorf’s tubes containing 500 µL of LB broth. Vanillin (25 mg/mL) was used as the positive control and the cultured bacteria as the negative control. Five-hundred (500) microliters of an overnight culture (OD 0.08–0.1 at 600 nm) was transferred to each Eppendorf’s tube. The tubes were incubated in an orbital shaking incubator (28 °C, 130 rpm) for 24 h. Following incubation, the Eppendorf’s tubes were then centrifuged at 13,000 rpm for 10 min to precipitate the insoluble violacein.

Thereafter, the supernatant was discarded and the pellet resuspended in 1 mL 100% DMSO and centrifuged as before to precipitate the cells. Afterwards, 1 mL of the solution was transferred into cuvettes for violacein quantification at 585 nm using a spectrophotometer (SpectraMax DU 720, Beckman Coulter, South Africa). The mean absorbance (OD585 nm) of the replicate samples was determined and the percentage inhibition calculated using the following Equation (1):

Percentage (%) inhibition = (ODcontrol-ODtest)**/**(ODcontrol) × 100 (1)

### 4.5. GC MS Analysis

GC-MS analysis was carried out by injecting one (1) µL of sample in the splitless mode at 250 °C onto an InertCap 5MS/NP capillary (30 m × 0.25 mm × 0.25 µm; GL Sciences, Tokyo, Japan). The ion source was operated at 250 °C, and the oven temperature was programmed as follows: 100 °C holding for 3 min and increased at 10 °C/min to 180 °C and ramped up to 200 °C at 20 °C/min for 0.5 min, with a final ramp up to 260 °C at 10 °C/min and a hold for 13 min. Helium was used as the carrier gas at a flow rate of 1.0 mL/min and a velocity of 32 cm/sec. Mass spectra were recorded between 50 to 600 m/z in the electron impact (EI) ionization mode at 70 eV with a scan speed of 2300. Compounds were tentatively identified by comparing the obtained mass spectra with those from published commercial libraries NIST11 (Gaithersburg, MD, USA) and Wiley (10th edition) (John-Wiley and Sons Inc., Hoboken, NJ, USA).

### 4.6. Molecular Docking of Selected Phytochemical Compounds

The molecular docking studies were conducted to determine the AQS potential and the mode of interactions with selected phytochemical compounds of *Calpurnia aurea* against the CviR protein of *C. violaceum* ATCC 12472 (PDB: 3QP1), as previously reported by Quecan et al. [42]. The 2-dimensional structure of the phytochemical compounds from *Calpurnia aurea* were obtained from PubChem or Wikipedia and drawn on Canvas 3.5 and exported to Maestro 11.5. The crystallized structures of the CviR protein of *C. violaceum* ATCC 12472 (PDB: 3QP1) and different ligands were obtained in the Protein Data Bank database (PDB). Prior to the docking experiments, chemically correct models of the ligands were generated using the ligprep of Schrodinger, and the receptor structure was done through a protein preparation wizard. Molecular docking was done using the Glide ligand docking module. Receptor grids were obtained using the Glide receptor. All docking calculations were performed using AutoDock 4.0. Grids (Schrodinger, LLC, New York, NY, USA) for the prepared protein were generated using the protein grid generation module. Further modifications included removing of H_2_O and metals before optimizing the hydrogen bonds and forcing minimization. The generated scores mimic the potential energy change when the protein and the compound come together based on hydrogen bonds, metal ions, and steric interactions. Lower scores (more negative) correspond to higher binding affinities. The best score of the docking of each compound was selected, allowing the inspection of the binding sites of the CviR protein with each compound [42]. The standard AQS agents included quercetin and vanillin structures, as reported in the literature.

### 4.7. Antivirulence Activity of Plant Extracts Against P. aeruginosa

#### 4.7.1. Inhibition of Cell Attachment

The 96-well flat bottom polystyrene microtiter plates (Lasec, Johannesburg, South Africa) were used to evaluate the ability of plant extracts (MICs below or 1 mg/mL) to inhibit the biofilm formation using the modified crystal violet (CV) assay. Only two plant extracts that showed MIC values of 1 mg/mL were tested. Briefly, 100 μL of MH broth, 100 μL of each extract, and 100 μL of standardized bacterial suspension (OD600 = 0.08–0.1) were added into the wells in triplicate. One-hundred microliters of ciprofloxacin (0.06 mg/mL) (instead of the plant extract) were also added into the wells. A total volume of 300 μL of the sterile MH broth and bacterial culture were added as a blank and negative control, respectively. The plates were then incubated at 37 °C for 24 h.

Crystal violet (CV) staining procedure: following incubation, cell attachment was evaluated by the CV staining assay. The wells were washed three times with sterile distilled water to remove the contents. The remaining biofilm left on the walls of the wells were then oven-dried at 60 °C for 45 min. Following drying, the wells were stained with 100 μL of 1% crystal violet solution and incubated at room temperature for 15 min. The wells were then rinsed three times with sterile distilled water to remove the excess, unabsorbed stain. To destain the wells, 125 μL of ethanol was added to each well and gently swirled to dissolve the stain from the biofilm. Blank wells were used to zero the microplate reader before taking the OD readings [43]. The absorbance was determined at 585 nm using a SpectraMax Paradigm microplate reader (Molecular devices, Separations, South Africa). The percentage inhibition was quantified using Equation (1).

#### 4.7.2. Inhibition of Preformed Biofilm

Biofilm of *P. aeruginosa* was developed for 8 h before being exposed to the plant extracts. An aliquot of 100 μL of Mueller Hinton broth and 100 μL of standardized bacterial suspension (OD 600 = 0.08–0.1) were added in each well. A total volume of 300 μL of bacterial culture and sterile MH broth were added in their three individual wells and were used as the negative control and as a blank, respectively. The plates were incubated at 37 °C for 8 h (under static conditions). After the 8 h incubation, 100 μL of each plant extract and ciprofloxacin (positive control) were transferred into their three individual wells. The plates were then incubated further at 37 °C for 24 h. Following incubation, CV staining procedure was followed as mentioned in Section 4.7.1, and the percentage inhibition was calculated.

#### 4.7.3. Confocal Laser Scanning Microscopy

Biofilm viability was assessed using confocal laser scanning microscopy (CSLM Carl Zeiss Microscopy, Jena, Germany). Biofilm of *P. aeruginosa* were grown on glass pieces (1 × 1 cm) placed in 24-well polystyrene plates and incubated at 37 °C for 8 h. After 8 h incubation, the preformed biofilm was supplemented with plant extracts and incubated further for 24 h. The adherent biofilm was gently washed with sterile distilled water, then stained with live/dead a backlight viability kit consisting of Syto 9 and propidium iodide (PI) and incubated for 15 min in the dark. After staining, the plates were washed again. SYTO 9 fluorescence was detected using a Zeiss LSM 510 (Carl Zeiss Microscopy, Jena, Germany) confocal laser-scanning microscope by excitation at 488 nm, and the emission was collected with a 500–530-bandpass filter [44].

#### 4.7.4. Swarming Motility Assay

The medium that was prepared for this assay was nutrient broth (0.8%, *w*/*v*), supplemented with glucose (3%, *w*/*v*) and agar of 0.5% (*w*/*v*). An aliquot (2 µL) of each plant extract (1 mg/mL) was mixed with 2 µL of an overnight culture (standardized at 600 nm OD = 0.08–0.1) and spotted on the nutrient agar plate. Ciprofloxacin and 1% DMSO were used as positive and negative controls, respectively. The plates were then incubated at 37 °C for 24 h–48 h. The zone diameters (mm) were measured to assess the swimming motility.

#### 4.7.5. Swimming Motility Assay

The swimming assay was performed by using swimming media according to Murray et al. [45], with slight modifications (1% tryptone, 0.5% NaCl, and 0.5% agar). The plates were inoculated with 2 μL of (OD 600 = 0.08–0.1) *P. aeruginosa* ATCC 9721 and incubated for 24–48 h at 37 °C. The diameter of the turbid zone (mm) was then measured and compared to the negative and positive controls.

#### 4.7.6. Statistical Analyses

Results unless otherwise stated were presented as mean ± standard deviations of three replicates. Generalized linear model (GLM) using PROC GLM in SAS was used to test if extracts have an effect on the initial attachment or biofilm formation. GLM was also used to test the inhibition and efficacies of different concentrations to the violacein formation. Means were separated using the least significant difference (LSD) method. All statistical analyses were carried out using Statistical Analysis System (SAS) program version 9.4, Stats Inc., 100 SAS Campus drive, Cary NC, USA, and *p*-values less than or equal to 0.05 were regarded as significant.

## 5. Conclusions

The findings from this study confirms that *Calpurnia aurea* plant extracts possess antipathogenic potential and are able to interfere with the QS system of *Pseudomonas aeruginosa*. The phytochemical compounds pentadecanol, dimethyl terephthalate, terephthalic acid, and methyl mannose identified from the plant suggested potent binding interactions and efficiency with the CviR protein active binding site of *Chromobacterium violaceum*. Further, the plant extract decreased virulence factors such as swimming and swarming and the biofilm by intervening with quorum sensing (QS), thus presenting the potential of these extracts in the development of antipathogenic drugs. The study serves as a preliminary to further studies aimed at determining the potential effect of multiple chemical compounds present in the ethanolic extracts of *Calpurnia aurea* that have the ability to interrupt the QS in *P. aeruginosa.* This study contributes to the search and prospecting for antibacterial resistance from using plant extracts used in folk medicine.

## Figures and Tables

**Table 1:** Minimum inhibitory concentration (MIC) mg/mL for plant extracts tested against the bacteria *Pseudomonas aeruginosa*.

Plant Species	Extracting Solvents Ethanol Acetone Ethyl Acetate		
*Calpurnia aurea*	1.56	1.56	6.25
*Leonotis ocymifolia*	6.25	6.25	6.25
*Moringa oleifera*	6.25	6.25	6.25
Positive and negative controls			
Ciprofloxacin	0.06		
1% DMSO	≥6.25		

**Table 2:** Qualitative anti-quorum sensing (AQS) activity against a biomonitor strain of *Chromobacterium violaceum C. aurea* extracts, where Ca.A = Acetone, Ca.E = Ethanol, and Ca.Y = Ethyl acetate extracts, respectively. R = Resistant, I = Intermediate, and S = Susceptible.

Zone Diameters (mm) and Associated Susceptibility Phenotypes			
*Chromobacterium violaceum* ATCC 31532			
Concentration (mg/mL)	Ca.A	Ca.E	Ca.Y
0.5	0.00 (R)	0.00 (R)	0.00 (R)
1	8.00 (R)	8.00 (R)	0.00 (R)
1.5	9.00 (R)	8.25 (R)	0.00 (R)
2	10.00 (R)	11.00 (I)	0.00 (R)
2.5	11.00 (I)	12.00 (S)	9.00 (R)
Positive and Negative Controls			
Ciprofloxacin (5 µg/mL)	29.30 (S)		
DMSO (1%)	0.00 (R)		

**Table 3:** Chemical components tentatively identified from ethanolic extracts of *Calpurnia aurea* using a gas chromatograph-mass spectrometer (GC-MS).

Peak	Ret. Time	Name	MS Fragmentation	Chemical Class	Area%
1	8.878	n-Teteradecane	198, 169, 141, 113, 99	Hydrocarbon	0.75
2	11.303	1,2-Benzenedicarboxylic acid	293, 167, 149, 127	Organic acid	2.76
3	11.921	1,4-Benzenedicarboxylic acid diethyl ester	222, 194, 177, 149	Organic acid ester	20.49
4	12.141	Methyl mannose	194, 177, 145, 115, 87	Sugar	15.76
5	13.301	n-Nonadecane	268, 196, 169, 141, 131, 99	Hydrocarbon	1.12
6	13.713	Phthalic acid undecene-undecyl ester	321, 304, 271, 167, 149, 132	Organic acid ester	3.79
7	14.115	Tetramethyl-2-hexadece-nol	296, 193, 138, 123, 96, 81	Alcohol	0.83
8	14.500	Beta-H-pregna	288, 165, 151, 125, 113, 125, 97, 57	Steroid	0.41
9	16.159	Terephthalic acid, ethyl isobutyl ester	250, 233, 195, 177, 149, 121, 84	Organic acid ester	3.96
10	16.243	Pentadecanol	210, 182, 168, 154, 140, 125, 111, 97, 83	Alcohol	14.54
11	18.322	Hexadecanol	224, 16, 168, 154, 139, 125, 111, 97	Alcohol	0.63
12	19.132	Octadecanol	252, 224, 210, 196, 182,168, 153, 139,125, 111, 97	Alcohol	1.70
13	20.055	2-(1*H*-indol-3-yl)acetic acid	334, 277, 253, 213, 199, 183	Organic acid	1.06
14	22.170	Octadecanal	268, 250, 224, 222, 208, 194,182	Aldehyde	1.01
15	25.310	Eicosanol	298, 280, 253, 167, 139, 125	Alcohol	12.12
16	27.085	Stigmasterol	412, 351, 300, 271, 255, 159	Steroid	0.65
17	28.973	Tetracosanal	352, 334, 306, 278, 250, 208	Aldehyde	3.94

**Table 4:** Binding analysis of 10 selected compounds identified from ethanolic extracts of *Calpurnia aurea.*

Compound	Total Energy	Docking Score	Glide Energy	H Bond
Pentadecanol	3.860	−3.758	−32.825	−0.358
Dimethyl terephthalate	6.895	−5.486	−34.901	−0.237
Phthalic acid	-	-	-	-
Eicosanol	-	-	-	-
Tetracosanal	-	-	-	-
Terephthalic acid	24.218	−6.186	−26.550	−0.385
Didecyl phthalate	-	-	-	-
Methyl mannose	9.681	−7.000	−37.276	−2.598
Vanillin	14.223	−4.880	−25.901	−0.872
Quercetin	25.683	−10.613	−44.877	−2.643

## Abbreviations

The following abbreviations were used in this manuscript:

3-oxo-C12 HSL	N-(3-oxo-dodecanoyl)-L-Homoserine lactone
AHL	acylhomoserine lactone
AQS	anti-quorum sensing
ATCC	American Type Culture Collection
c-di-GMP	cyclic dimeric guanosine monophosphate
cAMP	cyclic adenosine monophosphate
C4-HSL	N-butanoyl-L-homoserine lactone
DCM	dichloromethane
DMSO	dimethyl sulfoxide
EtAC	Ethyl acetate
HPTLC	high-performance thin layer chromatography
INT	*p*-iodonitrotetrazolium violet
LB	Luria Bertani
MIC	minimum inhibitory concentration
GC-MS	gas chromatography-mass spectrometry
QS	quorum sensing
QSI	quorum sensing inhibition
R	resistant
LPS	liposaccharides
MH	Muller Hinton
MS	mass spectrometry
NaCl	sodium chloride
CV	*Chromobacterium violaceum*
